# Supplementary material for: Salt-Related Knowledge, Attitudes, and Behaviors and Their Relationship with 24-Hour Urinary Sodium Excretion in Chinese Adults
Source: Nutrients. 2022 Oct 20;14(20):4404. doi: 10.3390/nu14204404 (PMC9611823; doi:10.3390/nu14204404)
Supplement: Supplementary file 1 [file nutrients-14-04404-s001.zip › nutrients-1941214-supplementary.pdf]

Salt-related knowledge, attitudes, and behaviors and their relationship with 24-hour urinary sodium excretion in Chinese adults

Supplementary Materials:

Table S1. Univariate and multivariate analysis (regression coefficient and *p*-value) of Knowledge score

Variables	Univariate analysis		Multivariate analysis	
	β (95% CI)	*p*–Value	β (95% CI)	*p*–Value
Location				
Urban	ref		ref	
Rural	–3.323(–3.621, –3.024)	<0.001	–0.876(–1.401, –0.395)	0.001
Sex				
Male	ref		ref	
Female	–0.316(–0.593, –0.039)	0.025	0.329(0.101, 0.556)	0.005
Age				
18–44	ref		ref	
45–59	–2.242(–2.554, –1.929)	<0.001	–0.219(–0.518, 0.059)	0.134
≥60	–3.464(–3.798, –3.131)	<0.001	–0.548(–0.893, –0.225)	0.001
Education				
Low	ref		ref	
Medium	3.255(2.990, 3.519)	<0.001	3.008(2.732, 3.284)	<0.001
High	7.483(7.201, 7.765)	<0.001	6.810(6.488, 7.136)	<0.001
Hypertension				
No	ref		ref	
Old diagnosed	–1.050(–1.448, –0.652)	<0.001	–0.053(–0.385, 0.281)	0.757
New observed	–1.750(–2.098, –1.402)	<0.001	–0.212(–0.517, 0.095)	0.156

Table S2. Univariate and multivariate analysis (regression coefficient and *p*-value) of attitude score

Variables	Univariate analysis		Multivariate analysis	
	β (95% CI)	*p*–Value	β (95% CI)	*p*–Value
Location				
Urban	ref		ref	
Rural	–1.425(–1.661, –1.190)	<0.001	–0.827(–1.124, –0.528)	0.003
Sex				
Male	ref		ref	
Female	1.045(0.835, 1.255)	<0.001	1.215(1.006, 1.423)	<0.001
Age				
18–44	ref		ref	
45–59	–0.856(–1.103, –0.608)	<0.001	–0.288(–0.575, –0.046)	0.030
≥60	–0.926(–1.190, –0.662)	<0.001	–0.223(–0.559, 0.056)	0.147

Education				
Low	ref		ref	
Medium	0.672(0.428, 0.915)	<0.001	0.774(0.524, 1.030)	<0.001
High	1.979(1.719, 2.239)	<0.001	1.776(1.483, 2.078)	<0.001
Hypertension				
No	ref		ref	
Old diagnosed	–0.489(–0.797, –0.182)	0.002	0.045(–0.261, 0.349)	0.772
New observed	–0.179(–0.447, 0.090)	0.192	0.415(0.134, 0.696)	0.004

Table S3. Univariate and multivariate analysis (regression coefficient and *p*-value) of behavior score

Variables	Univariate analysis		Multivariate analysis	
	β (95% CI)	*p*–Value	β (95% CI)	*p*–Value
Location				
Urban	ref		ref	
Rural	–1.175(–1.442, –0.907)	<0.001	0.650(0.059, 1.197)	0.024
Sex				
Male	ref		ref	
Female	1.128(0.890, 1.365)	<0.001	1.195(0.965, 1.425)	<0.001
Age				
18–44	ref		ref	
45–59	0.801(0.525, 1.077)	<0.001	0.916(0.626, 1.206)	<0.001
≥60	2.094(1.800, 2.389)	<0.001	2.086(1.751, 2.423)	<0.001
Education				
Low	ref		ref	
Medium	–0.801(–1.078, –0.524)	<0.001	0.027(–0.253, 0.305)	0.851
High	–0.036(–0.333, 0.261)	0.812	0.685(0.357, 1.012)	<0.001
Hypertension				
No	ref		ref	
Old diagnosed	–0.100(–0.446, 0.247)	0.573	0.340(–0.676, –0.003)	0.048
New observed	0.444(0.142, 0.747)	0.004	–0.242(–0.551, 0.068)	0.126
